# Adrenocorticotropic Hormone Suppresses Gonadotropin-Stimulated Estradiol Release from Zebrafish Ovarian Follicles

**DOI:** 10.1371/journal.pone.0006463

**Published:** 2009-07-31

**Authors:** Derek Alsop, Jennifer S. Ings, Mathilakath M. Vijayan

**Affiliations:** Department of Biology, University of Waterloo, Waterloo, Ontario, Canada; University of Alabama, United States of America

## Abstract

While stress is known to impact reproductive performance, the pathways involved are not entirely understood. Corticosteroid effects on the functioning of the hypothalamus-pituitary-gonadal axis are thought to be a key aspect of stress-mediated reproductive dysfunction. A vital component of the stress response is the pituitary secretion of adrenocorticotropic hormone (ACTH), which binds to the melanocortin 2 receptor (MC2R) in the adrenal glands and activates cortisol biosynthesis. We recently reported MC2R mRNA abundance in fish gonads leading to the hypothesis that ACTH may be directly involved in gonadal steroid modulation. Using zebrafish (*Danio rerio*) ovarian follicles, we tested the hypothesis that acute ACTH stimulation modulates cortisol and estradiol (E_2_) secretion. ACTH neither affected cortisol nor unstimulated E_2_ release from ovarian follicles. However, ACTH suppressed human chorionic gonadotropin (hCG)-stimulated E_2_ secretion in a dose-related manner, with a maximum decrease of 62% observed at 1 I.U. ACTH mL^−1^. This effect of ACTH on E_2_ release was not observed in the presence of either 8-bromo-cAMP or forskolin, suggesting that the mechanism(s) involved in steroid attenuation was upstream of adenylyl cyclase activation. Overall, our results suggest that a stress-induced rise in plasma ACTH levels may initiate a rapid down-regulation of acute stimulated E_2_ biosynthesis in the zebrafish ovary, underscoring a novel physiological role for this pituitary peptide in modulating reproductive activity.

## Introduction

It is well established that stress has a negative impact on reproductive processes in animals. Although the mechanisms are far from clear, the effects of stress are thought to be due to interactions of the hypothalamic-pituitary-adrenal (HPA) axis with the HP-gonadal (HPG) axis. For instance, corticotropin releasing factor (CRF), a key hypothalamic neurohormone that activates the HPA signaling cascade, also suppresses the release of hypothalamic gonadotropin-releasing hormone (GnRH) [1]. While corticosteroid is essential in order for animals to recover from exposure to a stressor, this steroid also impacts the HPG axis at a number of sites, depending on the species, sex, and the magnitude and duration of this plasma hormonal response [2], [3]. For instance, cortisol inhibits GnRH pulsatility [4], and decreases gonadotropin release [follicle stimulating hormone (FSH) and luteinizing hormone (LH)] [5], [6] from the pituitary. In the testes, cortisol suppresses testosterone production (reviewed by Hu et al. [7]) by reducing LH responsiveness, including downregulation of LH receptors [5].

In fish, cortisol decreased 11-keto testosterone production (the primary androgen in teleosts) [8], [9], but did not affect ovarian estradiol (E_2_) production in three species of fish [12]. However, cortisol treatment decreased hepatic expression of estrogen receptors (ER), vitelline envelope protein-β and vitellogenin [10], [11]. The latter two proteins are synthesized in the liver in response to ER activation and incorporated into the developing oocytes. These studies demonstrate that activation of the HPA axis (specifically CRF and cortisol) can impact reproductive performance by targeting multiple sites along the HPG axis. However, to our knowledge there has been no investigation linking adrenocorticotropic hormone (ACTH) signaling with reproductive function.

ACTH is acutely released from the pituitary gland in response to stressor-induced CRF stimulation and is the primary secretagogue for adrenal cortisol biosynthesis. In mammals, ACTH has been shown to stimulate cortisol production in extra-adrenal tissues, including the eye [13] and hair follicle [14]. Also, ACTH stimulates sex steroid production in neonatal rat testes [15], [16]. Recently, a real time quantitative PCR (qPCR)-based survey of MC2R gene expression in a teleost, the rainbow trout (*Oncorhynchus mykiss*), showed a high number of transcripts in the interrenal tissue (homolog of the mammalian adrenal gland) as well as the ovary and testis [17]. This led us to hypothesize that ACTH may modulate gonadal steroidogenesis in teleosts. To this end, we tested the actions of ACTH on ovarian cortisol and E_2_ secretion using the well characterized zebrafish ovarian follicle model [18], [19].

## Results

### Expression of MC2R

**Figure 1:**
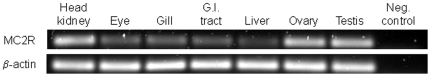
Tissue-specific melanocortin 2 receptor (MC2R) gene expression. Expression of melanocortin 2 receptor (MC2R) and β-actin in adult zebrafish tissues as determined with RT-PCR. Products were amplified (MC2R-36 cycles, β-actin- 32 cycles) from total RNA extracts. Images are representative of the results observed from three to five fish. Water replaced cDNA in the negative (Neg.) control treatments.

In zebrafish adults, transcripts for MC2R were most abundant in the head kidney (interrenal tissue), ovary and testis (Fig. 1). There were fewer transcripts in the eye, gill, gastrointestinal (G.I) tract and liver (Fig. 1).

### Ovarian follicle steroid production

#### Cortisol

**Figure 2:**
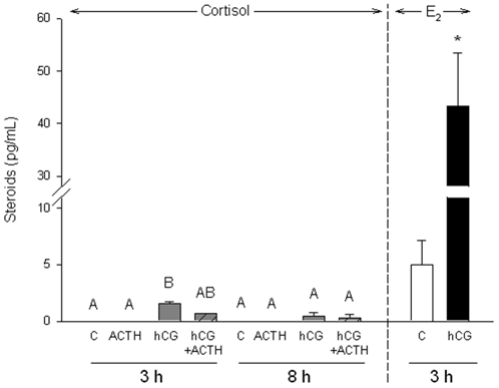
Temporal (3 or 8 h incubations) cortisol production by zebrafish ovarian follicles. Cortisol (grey bars) is not detected in the culture media of control or ACTH treated follicles, but is detected in the human chorionic gonadotropin (hCG, 10 I.U./mL) and hCG plus adrenocorticotropic hormone (ACTH, 1.5 I.U./mL; hatched bars) treatments. Control (open bar) and hCG-stimulated (black bar) estradiol (E_2_) media concentrations after 3 h are provided as a reference. Values represent mean ± SEM (*N* = 3; each *N* is a pool of follicles from three fish). Treatments with different letters are significantly different, as determined by one-way ANOVA at each time point followed by Student-Newman-Keuls test for multiple comparisons (P<0.05). An asterisk (*) with the E_2_ measurements indicates a significant difference determined by a Student's *t* test (P<0.05).

Cortisol was not detected in media from control and ACTH-treated ovarian follicles after 3 h or 8 h of incubation (Fig. 2). Cortisol was detected in follicles treated with human chorionic gonadotropin (hCG), although the levels were only significantly different from the control at 3 h of incubation (Fig. 2).

#### Estradiol

**Figure 3:**
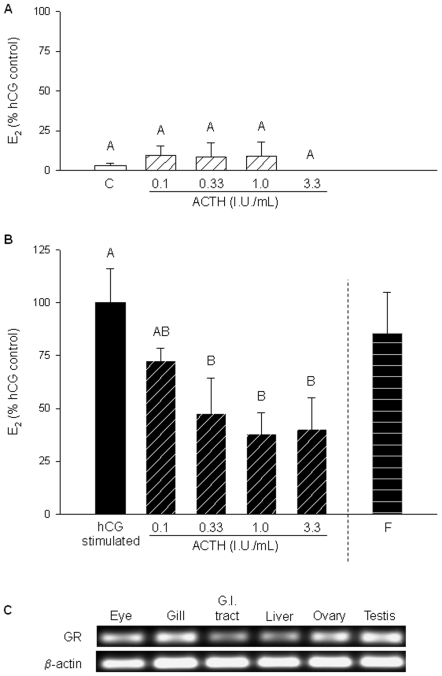
Effect of ACTH on estradiol (E_2_) production by zebrafish ovarian follicles. E_2_ production by follicles after 1.5 h incubations under A) basal conditions (no hCG; open bars) with a range of ACTH concentrations (0 to 3.33 I.U./mL; hatched bars), and B) hCG treatment (10 I.U./mL; black bars) and a range of ACTH concentrations (hatched bars). Additionally, the effect of 500 nM (181 ng/mL; horizontal striped bar) cortisol (F) on hCG-induced E_2_ production was also tested. Values represent mean ± SEM (*N* = 4, with each *N* representing a pool of follicles from three different fish). Treatments with different letters are significantly different (one-way repeated measures ANOVA followed by Student-Newman-Keuls test for multiple comparisons; P<0.05). The effects of cortisol on E_2_ production was analyzed using a Student's *t* test. C) Expression of the glucocorticoid receptor (GR) and β-actin in adult zebrafish tissues as determined with RT-PCR (GR-36 cycles, β-actin- 32 cycles). Images are representative of the results observed from three fish.

**Figure 4:**
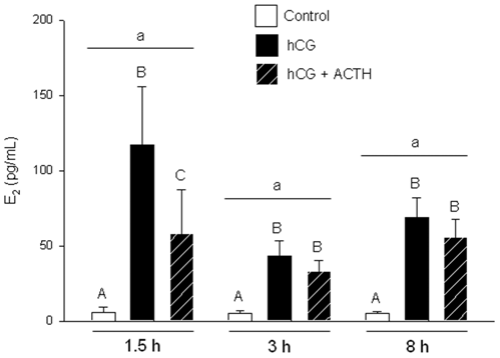
Temporal E_2_ production by zebrafish ovarian follicles. Temporal E_2_ production (1.5 h, 3 h or 8 h incubations) by zebrafish ovarian follicles in control (open bars), human chorionic gonadotropin (hCG; 10 I.U./mL; black bars) and hCG plus adrenocorticotropic hormone (ACTH; 1 I.U./mL at 1.5 h, 1.5 I.U./mL at 3 h and 8 h; hatched bars) treatments. Each time point is a separate experiment using follicles from different fish. Values represent mean ± SEM (*N* = 3 or 4, where each *N* is a pool of follicles from three different fish). Time points with the same lower-case letter are not different, while treatments within a given time that are different are indicated by different upper-case letters (two-way ANOVA; P<0.05).

**Figure 5:**
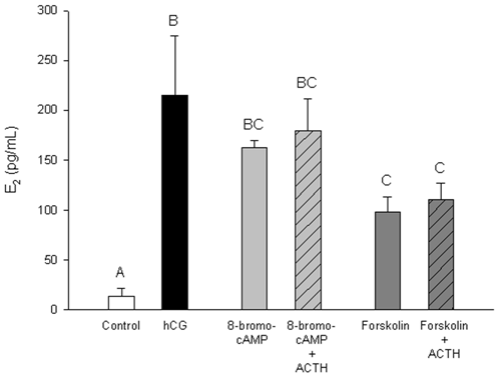
Estradiol (E_2_) production by zebrafish ovarian follicles in response to 8-bromo-cAMP and forskolin. Follicles were exposed for 1.5 h to basal conditions (open bar), hCG (10 I.U./mL; black bar), 8-bromo-cAMP (0.5 mM; grey bar), 8-bromo-cAMP and ACTH (1.0 I.U./mL; hatched bar), forskolin (10 µM; dark grey bar) or forskolin and ACTH (1.0 I.U./mL; hatched bar). Values represent mean ± SEM (*N* = 3 pools of follicles from three different fish). Treatments with different letters are significantly different (one-way ANOVA followed by Student-Newman-Keuls test for multiple comparisons; P<0.05).

Follicular estradiol (E_2_) secretion increased 9 to 35-fold by hCG treatment (10 I.U./mL) compared to the control group (in the absence of hCG; Figs. 2, 3, 4 and 5). A range of ACTH concentrations alone did not alter follicular E_2_ secretion (no hCG; Fig. 3A). However, ACTH significantly decreased hCG-stimulated E_2_ secretion in a dose-related manner (Fig. 3B). Maximum inhibition occurred at 1.0 IU ACTH/mL, which resulted in a 62% decrease in media E_2_ levels compared to hCG treatment alone (Fig. 3B). Cortisol did not inhibit hCG-stimulated E_2_ secretion, despite the expression of the glucocorticoid receptor (GR) in whole ovary samples (Fig. 3C).

Temporal E_2_ accumulation in the media was examined by comparing 1.5 h, 3 h and 8 h incubation experiments (Fig. 4). Each time point was a separate experiment performed with follicles from different fish. Total media E_2_ concentrations for a given treatment were not different between time points, or in other words, E_2_ concentrations did not increase after 1.5 h (Fig. 4). However, the magnitude of change between control and hCG-stimulated treatments was greatest at 1.5 h, and the effect of ACTH on hCG-stimulated E_2_ production was only significant at 1.5 h (Fig. 4).

8-bromo cAMP and forskolin both stimulated E_2_ secretion above basal levels (Fig. 5). Forskolin- but not 8-bromo cAMP-induced E2 production was significantly lower compared to hCG-stimulated E2 levels (Fig. 5). ACTH had no effect on either 8-bromo cAMP- or forskolin-induced E_2_ secretion in zebrafish ovarian follicles (Fig. 5). E_2_ secretion over 1.5 h was not altered by treatment with 0.1% dimethyl sulfoxide, the carrier for forskolin (data not shown).

## Discussion

**Figure 6:**
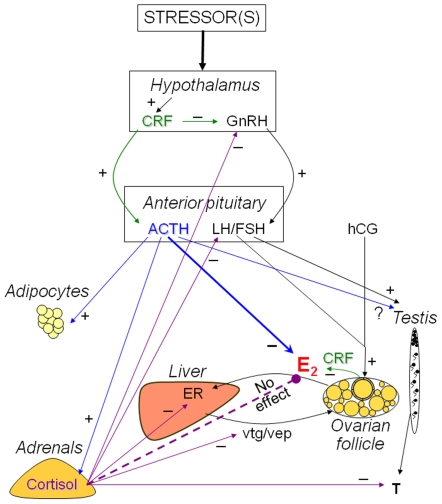
Overview of the impact of the stress axis (hypothalamus-pituitary-adrenal axis) on the reproductive axis (hypothalamus-pituitary-gonadal axis). Human chorionic gonadotopin (hCG) was used as a luteinizing hormone receptor agonist to stimulate estradiol (E_2_) secretion from ovarian follicles. Corticotropic releasing factor (CRF, green arrows) inhibits GnRH within the hypothalamus [1] and steroidogenesis in the ovary [31]. Adrenocorticotropic hormone (ACTH, blue arrows) inhibits stimulated estradiol (E_2_) synthesis by ovarian follicles (present study; blue bold arrow) and stimulates lipolysis in adipocytes [22]. Cortisol (purple arrows) modulates reproductive process in the hypothalamus, pituitary and testis [5]. However, cortisol has no effect on E_2_ synthesis in zebrafish ovarian follicles (present study; purple dashed arrow). In fish, cortisol treatment decreases the expression of hepatic estrogen receptor (ER), vitellogenin (vtg) and vitelline envelope protein-β (vep) [10], [11]. See text for more examples.

The present study demonstrates a novel physiological role for ACTH in modulating sex steroid production during acute stress in fish (see Fig. 6). This extra-adrenal role for ACTH involves the suppression of hCG-stimulated E_2_ secretion from zebrafish ovarian follicles. The rapid elevation of plasma ACTH is a key response to acute stressor exposure, and is responsible for the stimulation of cortisol release from the adrenal glands (or interrenal tissue in teleosts) [17]. The cortisol response is evolutionarily conserved and is essential for the animal to metabolically cope with stress [20]. Although chronic cortisol exposures perturb reproductive performance (e.g. Carragher et al. [8]; Fig. 6), there appears to be no direct effect of acute cortisol exposure on the ovary and E_2_ secretion (present study, [12]). The downregulation of gonadotropin-stimulated E_2_ release by ACTH appears to be tissue-specific, and is distinct from the stimulatory effect of this pituitary peptide on cortisol biosynthesis in the adrenals [17].

ACTH inhibited hCG-stimulated E_2_ production from zebrafish ovarian follicles in a dose-related manner. The greatest inhibition was observed at 1.0 I.U. ACTH/mL (2.58 µM), similar to the concentration that maximally stimulated cortisol production from head kidney preparations in rainbow trout [17]. Media E_2_ levels reached their highest concentrations by 1.5 h. Further follicular secretion of E_2_ may have been inhibited by an ultra short-loop negative feedback by E_2_ in the ovary [21], which may explain why the suppressive effect of ACTH was temporally dependent and only observed at 1.5 h.

Although ACTH binds to all of the five known G-protein coupled melanocortin receptors [22], we hypothesize that ACTH is exerting its effects on follicular steroidogenesis via MC2R based on the high numbers of MC2R transcripts in the zebrafish ovary. This is also supported by our recent study that confirmed MC2R as the major signaling receptor for ACTH action in rainbow trout interrenal tissue [17]. The distribution of MC2R transcripts in zebrafish is similar to a recent tissue qPCR survey of MC2R in rainbow trout, which also found the greatest MC2R expression in the head kidney, ovary and testis [17].

The underlying cellular pathway of ACTH-induced inhibition of ovarian follicle E_2_ secretion is unknown. At the adrenals, ACTH binding to MC2R activates G-proteins that stimulate the rise of intracellular cAMP via adenylate cyclase. This in turn up-regulates the expression of genes encoding key protein involved in corticosteroid synthesis, including steroidogenic acute regulatory protein (StAR), P450 side chain cleavage (P450scc) and 11β-hydroxylase, leading ultimately to cortisol synthesis [23], [24]. Similarly, E_2_ synthesis in the ovary is stimulated by LH (or hCG) binding to the LH receptor [18], which in turn activates G-proteins, a rise in cAMP and the expression and activity of steroidogenic genes including StAR, 17β-hydroxysteroid dehydrogenase type 3 and aromatase [19]. The lack of effect of ACTH on 8-bromo-cAMP- or forskolin-induced E_2_ synthesis suggests the mechanism of ACTH inhibition is upstream of adenylate cyclase activation and cAMP signaling. One hypothesis is that this tissue-specific MC2R signaling in the ovary may involve an inhibitory G-protein, while in the adrenals it is coupled to a stimulatory G-protein. However, this remains to be tested.

ACTH did not stimulate cortisol production in zebrafish ovarian follicles, as it does in the adrenals. However, hCG treatment did elevate cortisol secretion, albeit at a lesser magnitude compared to ACTH and interrenal tissue [17]. This may account for the cortisol deposited in developing fish oocytes that is utilized by the embryo [25]. Cortisol may also act as an anti-inflammatory agent in the ovary to promote healing and repair after ovulation [26]. Indeed, the expression of GR in the ovary (present study, [27]) suggests a role for cortisol in follicular development and/or embryogenesis. The GR mRNA abundance in zebrafish oocytes supports a role for maternal GR transcripts in zebrafish early development [25].

In addition to its effects on ovarian steroidogenesis, ACTH has other extra-adrenal functions. Previous studies have shown that ACTH stimulates adipocyte lipolysis in non-primate mammals (Fig. 6; reviewed by [22]), which is thought to provide a source of energy in the face of a stressor. In addition, while ACTH is primarily known for stimulating cortisol secretion by the adrenals, it is also a secretagogue for cortisol in other tissues. For example, an HPA axis homolog (CRF, ACTH and cortisol) has been detected completely within the retinal epithelium of the eye [13], skin [28] and hair follicle [14], which respond to stressors such as UV light. Whether follicular E_2_ synthesis responds to circulating ACTH or ACTH that is synthesized within the ovary is unknown. However, CRF has been detected in the trout ovary [29], and in mammals, CRF inhibited ovarian follicular E_2_ synthesis [30], [31]. Whether CRF directly inhibits E_2_ in these cases or acts via stimulation of ACTH that in turn inhibits E_2_, is unknown.

What is the physiological significance of this ACTH-mediated E_2_ inhibition by ovarian follicles during acute stress in fish? E_2_ has multiple biological functions including, but not limited to, stimulation of reproductive tissue growth, ovulation and metabolism [32]. In fish, E_2_ also stimulates hepatic vitellogenin and extra-embryonic membrane protein synthesis for oocyte growth and development [33]. All these actions of E_2_ will lead to an increased energy demand, which will strain the already high energetic cost associated with reestablishment of homeostasis during stress adaptation. In the short-term, we hypothesize that the stressor-induced ACTH surge may assist with energy substrate re-partitioning during acute stress by 1) rapidly downregulating acute stimulated E_2_ synthesis by the ovary, and the associated E_2_-dependent energy demanding pathways, and 2) upregulating corticosteroid synthesis by the adrenals, and the associated energy demanding pathways, which are essential for stress adaptation. However, longer-term stressor exposure and the resultant sustained ACTH stimulation may lead to reduced reproductive performance due to suppression of E_2_ levels.

In summary, we demonstrate for the first time that ACTH suppresses gonadotropin-stimulated E_2_ production in zebrafish ovarian follicles. As plasma ACTH levels increase in response to stressor exposure, our results implicate a novel physiological role for this cortisol secretagogue in the modulation of reproductive function. We propose that while this ACTH action may be adaptive in the short-term in assisting with energy substrate reallocation to cope with stress, long-term ACTH stimulation may lead to reproductive dysfunction due to E_2_ suppression. While the mechanism of action of ACTH in suppressing E_2_ synthesis is unclear, future work will focus on characterizing the MC2R signaling pathway in fish gonads, including crosstalk between MC2R and LHR. It will also be interesting to test whether ACTH has an effect on testicular steroidogenesis, given the high number of MC2R transcripts expressed in the testis (Fig. 1, [17]). It remains to be seen if the role of ACTH in mammalian reproductive tissues is similar to that of fish, given the different reproductive strategies exhibited by mammals (viviparity) and fish (yolk; oviparity). However, the expression of MC2R in human ovary and testis [34], similar to zebrafish, suggests a role for ACTH in modulating gonadal function.

## Materials and Methods

### Animals

Adult zebrafish (*Danio rerio*) were purchased from a commercial supplier and maintained in a recirculating system (Aquatic Habitats, Apopka, FL, USA) at 28°C in well water (hardness = 400 mg CaCO_3_/L, pH = 8.0). Photoperiod was 12 h light/12 h dark. Fish were fed two or three times daily a mix of pellets (New Life Spectrum), spirulina flakes and bloodworms. All experiments were performed in accordance with the University of Waterloo Animal Care Committee and conformed to the guidelines of the Canadian Council on Animal Care.

### Receptor expression

The expression of MC2R, glucocorticoid receptor (GR) and β-actin were examined using RT-PCR in the head kidney, eye, gill, gastrointestinal tract (G.I. tract), liver, ovary and testis. Fish were euthanized with an overdose of MS222 (0.25 g/L) followed by spinal severance. Tissues were excised, immediately frozen on dry ice in microfuge tubes and stored at −80°C until processing. Total RNA was extracted with QIAzol Lysis Reagent (phenol and guanidine thiocyanate) and purified with an RNeasy Mini Kit (Qiagen; Mississauga, ON, Canada). Total RNA was treated with DNase (Qiagen) to remove genomic DNA and quantified using a Nanodrop spectrophotometer (Wilmington, DE, USA). First strand cDNA was synthesized using a commercial kit (MBI Fermentas; Burlington, ON, Canada), where 1 µg of total RNA was reverse transcribed in 20 µL using M-MuLV reverse transcriptase (40 U), oligo (dT)_18_ primers (0.5 µg), dNTPs (1 mM each) and a ribonuclease inhibitor (20 U) in a total volume of 20 µL.

PCR reactions were carried out with the following primers: β-actin F 5′-tgtccctgtatgcctctggt-3′, R 5′-aagtccagacggaggatgg-3′ (product size = 121 b.p.); MC2R F 5′-ctccgttctcccttcatctg-3′, R 5′-attgccggatcaataacagc-3′ (product size = 127 b.p.); GR F 5′-acagcttcttccagcctcag-3′, R 5′-ccggtgttctcctgtttgat-3′ (product size = 116 b.p.). Gene amplification consisted of an initial denaturing period of 95°C for 3 min, followed by cycles of: 1) denaturing at 95°C for 30 s, 2) annealing at 60°C for 30 s and 3) extension at 72°C for 30 s. The number of cycles were 32 for β-actin and 36 for MC2R and GR. This was followed by a 10 min extension period at 72°C. PCR reactions were fractionated in 1.5% agarose gels along with DNA molecular weight standards, stained with ethidium bromide and images were captured under UV light.

### In vitro follicle incubations

Follicular steroid secretion and steroid RIAs were determined using methods developed and validated by Ings and Van Der Kraak [19]. Briefly, female zebrafish were anesthetized (0.1 g MS222/L) and decapitated. Whole ovaries were removed and placed in Leibovitz -15 (L-15) media (Gibco, Grand Island, NY, USA) with antibiotics (penicillin and streptomycin) and antimycotic (amphotericin B) solution (Sigma-Aldrich) at room temperature. Follicles from three fish were separated and pooled for each *N*, then 60 or 70 stage 2 (previtellogenic) and stage 3 (vitellogenic) oocytes [35] were evenly distributed in a 24 well plate. At the start of the incubation, medium was removed and replaced by 800 mL of fresh medium containing 0.5 mM 3-isobutyl-1-methylxanthine (Sigma-Aldrich), which inhibits phosphodiesterases that degrade cAMP. Follicles were treated with a variety of compounds. In the first series of experiments, follicles were treated with human chorionic gonadotropin (hCG; 10 I.U./mL [19]) and/or ACTH (1.5 I.U./mL, porcine ACTH^1–39^) (Sigma-Aldrich). In the second series, follicles were treated with hCG (10 I.U./mL), ACTH (0.1 to 3.3 I.U./mL) or cortisol 500 nM (181.3 ng/mL). In the third series, follicles were treated with hCG (10 I.U./mL), ACTH (1.0 I.U./mL), forskolin (10 µM, dissolved in dimethyl sulfoxide) or 8-bromo cAMP (0.5 mM) (Sigma-Aldrich). Incubations were 1.5 h, 3 h or 8 h. At the end of the experiment, medium was removed and stored at −70°C until steroid analyses. All experiments were repeated with three or four independent samples.

#### RIAs

Cortisol and E_2_ (17β-estradiol) in the culture media were measured by radioimmunoassay (RIA) according to Ings and Van Der Kraak [19]. Antibodies were purchased from Medicorp (Montreal, PQ, Canada) and [^3^H]-steroids were obtained from Amersham (Baie d'Urfé, PQ, Canada).

### Statistical analysis

Data were initially screened for normality and homogeneity of variance prior to analysis of variance (ANOVA). The time course of E_2_ production was analyzed with a two-way ANOVA using time and treatment as factors. All other data were analyzed with a one-way ANOVA or one-way repeated measures ANOVA. This was followed by Student-Newman-Keuls test for multiple comparisons (SPSS) to determine significant differences among groups. The effects of cortisol on E_2_ production was analyzed using a Student's *t* test. Differences were considered significant if P<0.05.
